# Comparative Proteomic Insights into the Immune Response of *Conogethes punctiferalis* Challenged with *Beauveria bassiana*

**DOI:** 10.3390/insects16070667

**Published:** 2025-06-26

**Authors:** Shaohua Li, Zhiwei Kang, Xiangdong Li, Hailei Wei, Xiangchu Yin, Fangqiang Zheng, Fanghua Liu

**Affiliations:** 1 College of Plant Protection, Shandong Agricultural University, Tai’an 271018, China; lish1004@163.com (S.L.); zwkang@hbu.edu.cn (Z.K.); xdongli@sdau.edu.cn (X.L.); 2 College of Resources and Environment, Shandong Agricultural University, Tai’an 271018, China; 3 College of Life Science, Hebei University, Baoding 071002, China; 4 Institute of Plant Protection, Shandong Academy of Agricultural Sciences, Jinan 250100, China; 5 Institute of Agricultural Resources and Regional Planning, Chinese Academy of Agricultural Sciences, Beijing 100081, China; weihailei@caas.cn

**Keywords:** *Conogethes punctiferalis*, *Beauveria bassiana*, proteomic, insect immunity

## Abstract

The yellow peach moth (YPM), *Conogethes punctiferalis*, is an important agricultural insect pest causing severe damage to corn in eastern China. *Beauveria bassiana* is an effective, eco-friendly, and promising alternative agent for controlling this insect pest. However, insect immunity can limit the ability of fungal infections. In order to understand the immune response mechanism of YPM, a comparative proteomic analysis of non-infected and *B. bassiana*-infected larvae was conducted using the isobaric tags for relative and absolute quantification (iTRAQ) technique. On the basis of proteomic analysis, 4195 quantifiable proteins were identified from a total of 29,155 peptides. The functions of the identified proteins were annotated in four databases (GO, COG, KEGG, and IPR). A total of 132 immune-related proteins were screened, including 46 pathogen recognition proteins, 27 extracellular signal modulation proteins, and 59 immune effectors. Furthermore, 70 differentially expressed proteins (DEPs) were identified, including 57 up-regulated proteins and 13 down-regulated proteins. Among these, four DEPs were related to immunity, namely one defense protein and three peptidoglycan recognition proteins. Six randomly selected immune-related proteins associated with target genes were validated for qRT-PCR, and the results indicated that the accuracy and reliability of the proteome sequencing data were high. Taken together, the results enrich the fundamental knowledge of YPM immune responses to *B. bassiana* infection and provide a new insight into insect−pathogen interactions.

## Simple Summary

The yellow peach moth (YPM), *Conogethes punctiferalis*, is a polyphagous insect pest that has been reported to cause severe damage to many crop species in China. *Beauveria bassiana*, as a common and effective entomopathogenic fungus, is extensively used for the biocontrol of various insect pests. In this study, we carried out the proteomic analysis of YPM larvae infected with *B. bassiana* using the isobaric tags for relative and absolute quantification (iTRAQ) technique. The immune-related proteins were screened based on the proteome data. The differentially expressed proteins (DEPs) were screened and identified, and then these DEPs were subjected to functional enrichment analysis. The accuracy and reliability of the proteome sequencing data were validated by qRT-PCR. Our results offer a new insight into the immune responses of YPM larvae infected with *B. bassiana* at the protein level and contribute to understanding the immune mechanisms of YPM larvae in response to *B. bassiana* infection.

## 1. Introduction

The yellow peach moth (YPM), *Conogethes punctiferalis* (Lepidoptera: Crambidae), is an important polyphagous insect pest extensively distributed across subtropical and tropical Asia and Australia and can attack over 100 species of field crops, fruits, and vegetables [1,2]. In China, *Ostrinia furnacalis* is considered a highly destructive insect pest of corn, and yet YPM has replaced *O. furnacalis* as the dominant insect pest on corn in the Huang-Huai-Hai region in recent years [3,4]. Currently, the utilization of chemical insecticides is the most common method for controlling YPM populations [5]. Regrettably, the overuse of chemical insecticides can lead to undesirable consequences, including environmental pollution and insect resistance to chemical insecticides [6,7].

Entomopathogenic fungi have been developed as an eco-friendly alternative to chemical insecticides because of their specific mode of action and ability to infect a vast array of insect pests [8,9]. Among them, *Beauveria* and *Metarhizium* are widely applied for the biocontrol of diverse insect pests [10,11]. For example, the utilization of *Beauveria bassiana* in the management of *Dendrolimus punctatus* has become a successful biological control scheme in China [12]. A highly virulent strain of *Metarhizium pingshaense* had a mortality in excess of 86% against YPM larvae under laboratory conditions [13]. The potential suppression of YPM larvae by *B. bassiana* would make it a promising alternative agent for controlling the insect pest in a previous study [14]. In fact, the well-developed innate immunity of insects to entomopathogenic fungi greatly limits the development and application of fungal biopesticides [15]. Despite lacking adaptive immunity in insects, their powerful innate immune systems, consisting of cellular and humoral immunity, are still capable of eliminating microbial pathogenic infections [16,17]. Therefore, it is crucial to explore the immune mechanisms of insects for enhancing the efficiency of fungal infections.

Due to the rapid development of molecular biology technology, numerous studies on insect immunity have been reported based on genome, transcriptome, and proteome analyses [18,19,20]. The genome of YPM has recently been published, constituting invaluable information for exploring the evolutionary mechanism of the YPM immune system [21]. However, proteins participating in the YPM immune response have not yet been comprehensively characterized. Proteomic analysis is able to provide highly valuable insights into the changes to the proteins in response to a specific stimulus or stress [22]. The isobaric tags for relative and absolute quantification (iTRAQ) technique has recently been increasingly applied due to its high sensitivity and accurate quantification [23,24,25]. To obtain more information on the immune responses of YPM to *B. bassiana* infection, the present study firstly reports a comparative iTRAQ-based quantitative proteomic analysis of YPM larvae infected with *B. bassiana*, thereby providing new insights into the immune molecular mechanism of insect−pathogen interactions.

## 2. Materials and Methods

### 2.1. Insect Rearing

YPM larvae were fed on fresh corn kernels in an artificial climate chamber (RXZ-380C, Ningbo, Zhejing, China) under the conditions of 25 ± 1 °C, 70 ± 5% relative humidity, and a 14 h light (with 5000 lx)/10 h dark cycle. The trials were conducted using three-day-old 5th-instar larvae.

### 2.2. Fungal Infection

*B. bassiana* strain (ACCC30107) was cultured on potato dextrose agar (PDA) plates at 26 °C and 80% relative humidity. Mature conidia were collected and the suspensions were prepared using sterile phosphate-buffered saline (PBS, pH = 7.4). The concentration was adjusted to 1 × 10^5^ conidia/μL by the hemocytometer. Three-day-old 5th-instar larvae were inoculated with 2 μL conidial suspensions (1 × 10^5^ conidia/μL) or sterile PBS (as control) using a microinjector (Hamilton, Bonaduz, Switzerland). Each treatment or control group included 30 larvae. Each bioassay was performed in triplicate. According to a previous study [14], the whole body of YPM larvae was frozen in liquid nitrogen at 36 h post-infection for further use.

### 2.3. Total Protein Extraction

The whole YPM larvae were ground into powder in liquid nitrogen and immediately transferred to the pre-cooled centrifuge tubes. The PASP protein lysis buffer (100 mmol/L ammonium bicarbonate, 8 mol/L urea, pH = 8) was added and then vortexed and mixed thoroughly. The samples were subjected to 5 min of ultrasonication in an ice-water bath to ensure complete lysis. The lysate was centrifuged (12,000× *g*, 4 °C) for 15 min, and the supernatant was collected. The dithiothreitol (DTT, 10 mmol/L) was added to the mix for 1 h at 56 °C. The iodoacetamide (IAM) was then added and placed for 1 h at room temperature in a condition of complete darkness. The above mixture was precipitated with four times the volume of pre-cooled acetone at –20 °C for at least 2 h, and the precipitation was collected using a centrifuge (12,000× *g*, 4 °C) for 15 min. Subsequently, the precipitation was resuspended and washed with 1 mL of –20 °C pre-cooled acetone and collected again by centrifuging at 12,000× *g* for 15 min at 4 °C. The samples were air-dried, and the protein dissolving solution (8 mol/L urea, 100 mmol/L triethylammonium bicarbonate (TEAB), pH = 8.5) was added to dissolve protein precipitation.

### 2.4. Protein Quality Test

According to the method of the Bradford protein quantitative kit, Bovine serum albumin (BSA) standard protein solution was prepared. The concentration gradient ranged from 0 to 0.5 g/L. BSA standard protein solutions with different concentration gradients were prepared and added into a 96-well plate. The sample solutions were then diluted to different concentrations and also added into the above plate. The volume of each well was 20 µL, and each gradient was repeated three times. A volume of 180 μL Coomassie Brilliant Blue G-250 dye solution was quickly added into the plate and left for 5 min at room temperature. The absorbance at 595 nm was then detected. The standard curve was drawn according to the absorbance, and the concentration of the protein samples was then calculated.

### 2.5. iTRAQ Labeling

The protein samples were made up to 100 μL using the dissolution buffer (DB buffer) (8 mol/L urea, 100 mmol/L TEAB, pH = 8.5). Trypsin (1 μg/μL) and TEAB (100 mmol/L) buffer were added, mixed well, and the mixture was digested at 37 °C for 4 h. Formic acid was added, used to adjust the pH to <3, and mixed well. The samples were then centrifuged (12,000× *g*) for 5 min. The sample was gradually loaded onto the C18 desalting column and washed three times consecutively with the washing buffer (0.1% formic acid, 3% acetonitrile). A volume of 300 μL eluent solution (0.1% formic acid, 70% acetonitrile) was added, and then the eluents were collected and lyophilized. A volume of 20 μL of 1 mol/L TEAB buffer was added to reconstitute. Each sample was added 5.5 μL of iTRAQ labeling reagent and mixed with shaking for 2 h. After that, a volume of 100 μL of 50 mmol/L Tris-HCl (pH = 8) was added and used for terminating the reaction. The labeling samples were mixed at an equal volume, desalted, and then lyophilized.

### 2.6. Separation of Fractions

The mobile phases A (2% acetonitrile, 98% water, pH = 10) and B (98% acetonitrile) were prepared to achieve a gradient elution effect. Solution A with dissolved lyophilized powder was centrifuged (14,000× *g*, 4 °C) for 20 min. The samples were fractionated using a Waters BEH C18 (Waters Corporation, Milford, MA, USA) chromatographic column (4.6 × 250 mm, 5 μm) on the L-3000 HPLC system. A tube was collected every minute, combined into ten fractions, lyophilized, and dissolved by the addition of 0.1% (*v*/*v*) formic acid.

### 2.7. Liquid Chromatography and Mass Spectrometry (LC-MS/MS) Analysis

The shotgun proteomic analyses were carried out using an EASY-nLCTM 1200 UHPLC system (Thermo Fisher, Waltham, MA, USA) and a Q ExactiveTM HF-X mass spectrometer (Thermo Fisher, Waltham, MA, USA). Samples were added into a C18 Nano-Trap column (4.5 cm × 75 μm, 3 μm). The separation of peptides was achieved through the utilization of an analytical column (15 cm × 150 μm, 1.9 μm) employing a linear gradient elution technique (Table S1). The separated peptides were checked by means of a Q ExactiveTM HF-X mass spectrometer. The full scanning range was from 407 to 1500 *m*/*z*. The automatic gain control (AGC) target value was set at 3 × 10^6^, and the maximum ion injection time was 20 ms. The top 40 most abundant precursors were selected for fragmentation, followed by detection using MS/MS. The raw data used for MS detection was designated “.raw”.

### 2.8. Identification and Quantitation of Proteins

The resulting spectra of each run were searched individually in the protein database (761199-X101SC21043487-Z01-Conogethes_punctiferalis-customer.pep.fasta (38033 sequences)) using the Proteome Discoverer 2.2 software. The searched parameters were set as follows: a mass tolerance of 10 mg/L for precursor ions and a mass tolerance of 0.02 Da for the product ions. The Proteome Discoverer 2.2 software was used to filter the retrieval results. The peptide spectrum matches (PSMs) with over 99% credibility were identified, and credible proteins contained a minimum of one specific peptide. Only credible PSMs and proteins were retained and conducted with FDR less than 1%. The results of the protein quantitation were analyzed using a *t*-test. The differentially expressed proteins (DEPs) (the up-regulated expression protein: fold change (FC) ≥ 1.5, *p* value ≤ 0.05; the down-regulated expression protein: FC ≤ 0.67, *p* value ≤ 0.05) were screened and identified.

### 2.9. Functional Annotation and Enrichment Analysis

Gene Ontology (GO) and InterPro (IPR) functional annotation were performed using the IterProScan 5.0 software in the non-redundant protein database (Pfam, PRINTS, ProDom, SMART, ProSite, and PANTHER). Clusters of Orthologous Groups (COG) and Kyoto Encyclopedia of Genes and Genomes (KEGG) annotations were conducted by subjecting the identified proteins to BLAST 2.2.26 software comparison (blastp, *E* value ≤ 10^−4^), then filtering the BLAST results for each sequence and selecting the comparison with the highest score for annotation. The protein family and pathway analyses were conducted through COG and KEGG [26]. The cluster heatmap, GO, KEGG pathway, and IPR enrichment analyses of DEPs were performed [27]. The cloud tools at NovoMagic (https://magic-plus.novogene.com/#/tool/list, accessed on 7 September 2024) were used for data visualization.

### 2.10. qRT-PCR Validation

The total RNA of YPM larvae was extracted and reverse transcribed into cDNA according to the kit method (Tiangen, Beijing, China). Specific primers were designed by Primer Premier 6 software (Table S2), and ribosomal protein 49 (RP49) was selected as the internal reference gene. The reaction system with a volume of 20 μL, consisting of 10 μL SuperReal PreMix Plus (2×), 1 μL upstream and downstream primers, 1 μL cDNA template, and 7 μL RNase-free ddH_2_O, was used for qRT-PCR by Bio-Rad CFX96 Touch Real Time PCR Detection System (Bio-Rad, Hercules, CA, USA). The reaction conditions were as follows: pre-denaturation at 95 °C for 15 min, followed by 40 cycles of denaturation at 95 °C for 10 s and annealing/extension at 60 °C for 30 s. All samples were analyzed in triplicate and repeated thrice as independent biological replicates. The qRT-PCR data were calculated using the 2^−ΔΔCT^ method [28].

## 3. Results

### 3.1. Identification and Quality Control of Proteome

In the present study, a total of 62,669 (11.08%) matched spectra in the 565,469 total spectra and 29,155 peptides, 4197 identified proteins, and 4195 all quantifiable proteins were detected and identified (Table 1).

**Figure 1:**
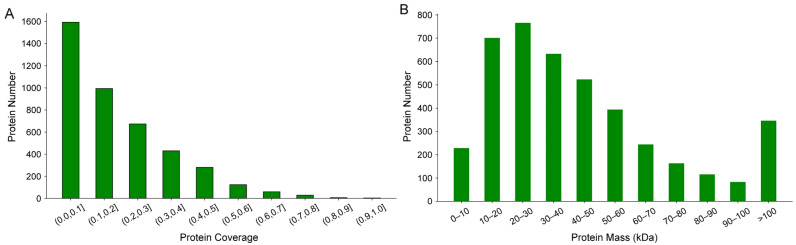
Distribution of protein coverage (**A**) and protein mass (**B**).

Quality control of proteome data was performed to ensure accuracy and reliability. The results showed that more than 90% of the proteins had a coverage between 0 and 0.5 (Figure 1A). In total, 766 proteins with a mass ranging from 20 to 30 kDa were identified, followed by 701, 633, and 523 proteins with a mass of 10–20, 30–40, and 40–50 kDa, respectively. The remaining 346 proteins had a mass greater than 100 kDa (Figure 1B).

### 3.2. Functional Annotation and Classification of Proteome

**Figure 2:**
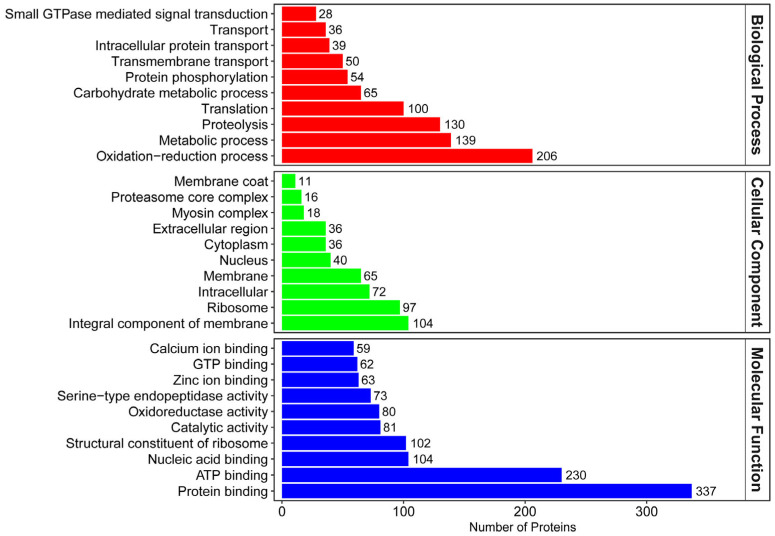
GO function annotation.

According to the GO annotations, a total of 2312 proteins were divided into three categories (Figure 2). In biological processes, the top three most frequent categories were oxidation–reduction process (206 proteins), metabolic process (139 proteins), and proteolysis (130 proteins). In cellular components, the top three most frequent categories were integral components of the membrane (104 proteins), intracellular (97 proteins), and ribosome (72 proteins). In molecular function, the top three most frequent categories were protein binding (337 proteins), ATP binding (230 proteins), and nucleic acid binding (104 proteins).

**Figure 3:**
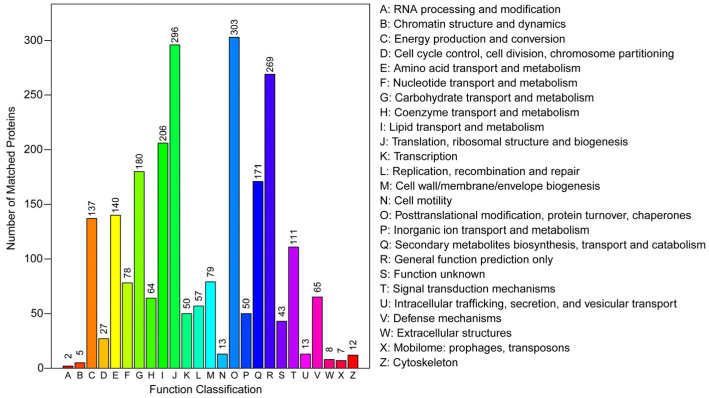
COG function annotation.

A total of 2122 proteins were classified into 25 categories based on the COG function classification (Figure 3). Among these, the largest group was posttranslational modification, protein turnover, and chaperones (303 proteins), followed by translation, ribosomal structure, and biogenesis (296 proteins), and general function prediction only (269 proteins).

**Figure 4:**
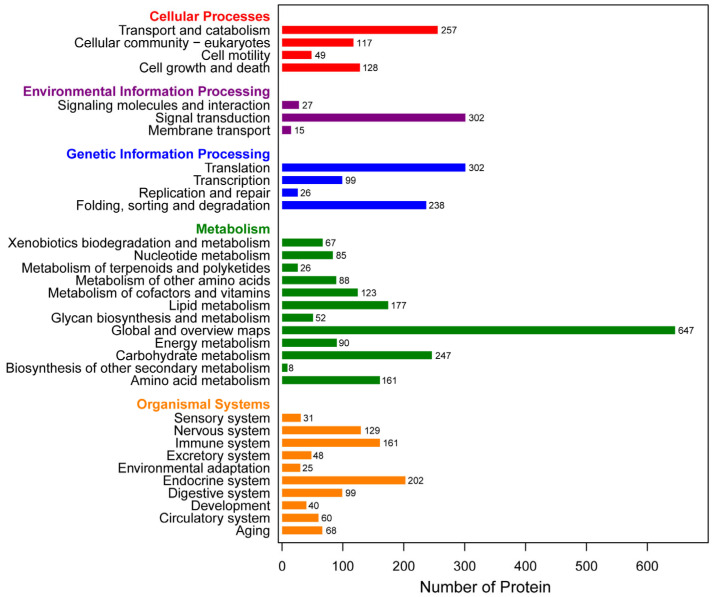
KEGG function annotation.

In total, 4107 proteins were mapped against the KEGG pathway and were categorized into five groups (Figure 4). The most enriched pathways were transport and catabolism (257 proteins), signal transduction (302 proteins), translation, global, and overview maps (647 proteins), and the endocrine system (202 proteins) in each category.

**Figure 5:**
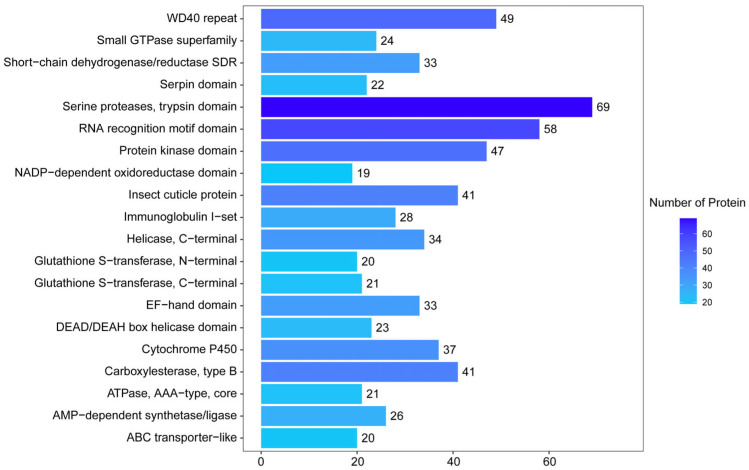
IPR function annotation.

The IPR analysis showed that a total of 3505 proteins were annotated, and the top three most frequent categories were serine protease, trypsin domain (69 proteins), RNA recognition motif domain (58 proteins), and WD40 repeat (49 proteins) (Figure 5).

### 3.3. Identification of the Immune-Related Proteins

A total of 132 immune-related proteins were screened and identified based on the YPM larval proteome data, including 46 pathogen recognition proteins, 27 extracellular signal modulation proteins, and 59 immune effectors (Table S3).

### 3.4. Statistics of DEPs

**Figure 6:**
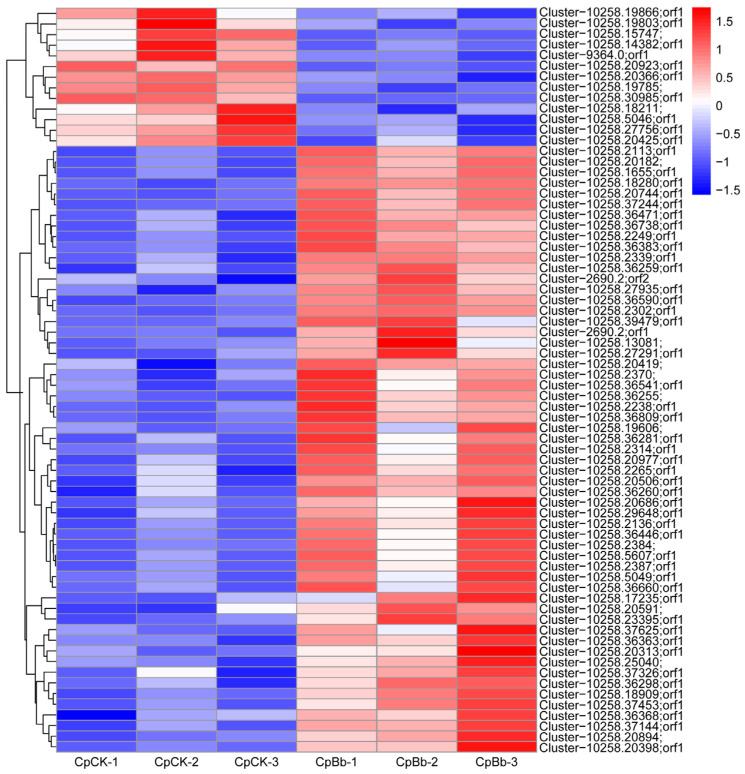
The cluster heatmap of DEPs.

The results of the differential protein screening showed that a total of 70 DEPs were identified, including 57 up-regulated proteins and 13 down-regulated proteins (Table 2). The cluster heatmap of DEPs is shown in Figure 6. After eliminating the undescribed proteins, 57 DEPs were identified, of which 47 were up-regulated and 10 were down-regulated (Table 3). Among these DEPs, four up-regulated DEPs were related to immunity, namely defense protein 3-like, peptidoglycan recognition protein B (PGRP-B), peptidoglycan recognition protein-like (PGRP), and peptidoglycan recognition protein LB-like (PGRP-LB).

### 3.5. Functional Enrichment Analysis of DEPs

**Figure 7:**
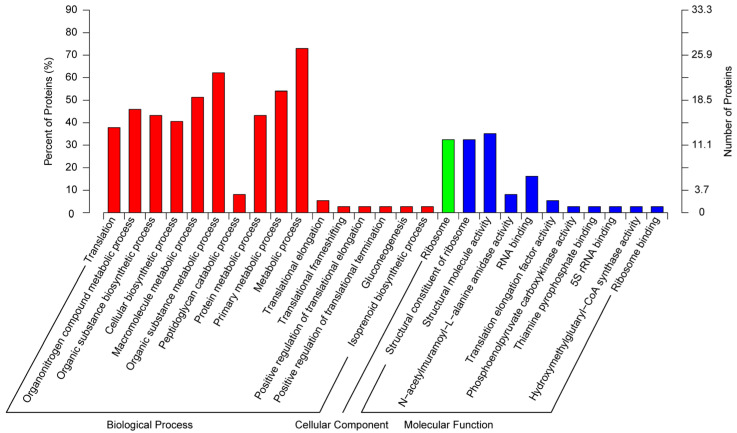
GO functional enrichment analysis of DEPs.

In total, 37 DEPs were enriched in GO enrichment analysis (Figure 7). In the biological process group, the DEPs were present in the metabolic process (27 DEPs), the organic substance metabolic process (23 DEPs), and the primary metabolic process (20 DEPs). In the cellular component group, the DEPs were mainly present in the ribosome (12 DEPs). In the molecular function group, more DEPs existed in the structural molecule activity (13 DEPs), structural constituent of ribosome (12 DEPs), and organic cyclic compound binding (11 DEPs).

**Figure 8:**
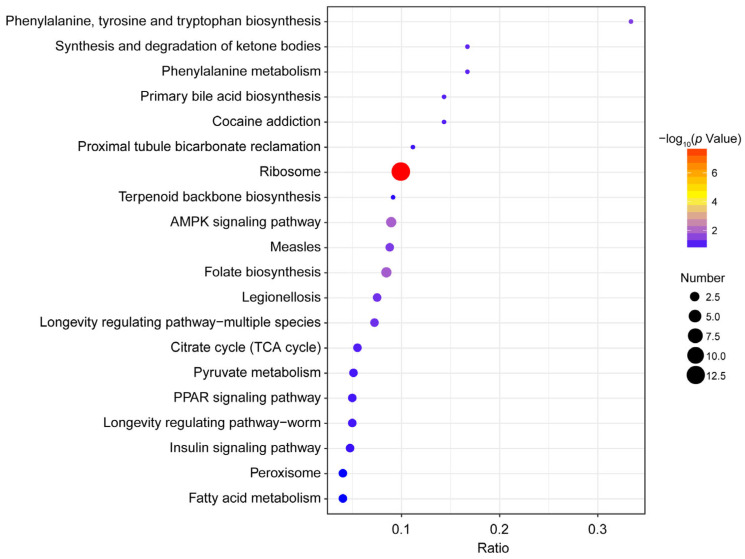
KEGG pathway analysis of DEPs.

Based on the KEGG pathway analysis, 29 DEPs were enriched, leading to the generation of 47 maps. The bubble chart shows the 20 most enriched KEGG pathways (Figure 8). The top three enriched pathways were ribosome (13 DEPs), AMPK signaling pathway (3 DEPs), and folate biosynthesis (3 DEPs), respectively.

**Figure 9:**
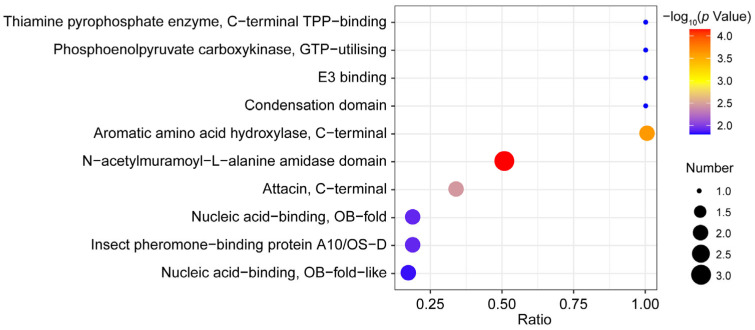
IPR enrichment analysis of DEPs.

A total of 55 DEPs were enriched according to IPR enrichment analysis. The bubble chart shows the 10 most enriched IPR (Figure 9). The top category was N-acetylmuramoyl-L-alanine amidase domain (3 DEPs).

### 3.6. Validation of Several DEPs by qRT-PCR

**Figure 10:**
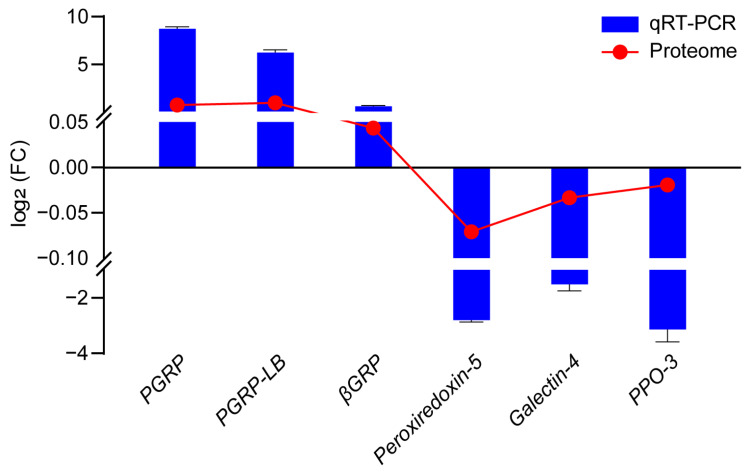
Verification of the proteome data.

To further verify the consistency of gene expression changes at both the mRNA and protein levels, six immune-related genes encoding proteins were selected for qRT-PCR analysis (Table S2 and Figure 10). The results revealed that the expression patterns of six genes at the mRNA level were consistent with those at the protein level, indicating that the accuracy of the proteome data was high.

## 4. Discussion

Transcriptomics or genomics are used to evaluate the messenger alone; by contrast, proteomics is a more direct means of describing molecular reactions, which can offer extremely useful information on the changes in proteins under various conditions and factors [22,25]. With the rapid development of omics techniques, iTRAQ has become a powerful and effective technique in proteomics due to its high sensitivity and accurate quantification [23,29]. Proteins are the pivotal final products of cells, which are capable of performing physiological functions [25]. The detection of changes at the protein level is capable of offering more direct evidence to study the immune mechanisms of insects. Here, a comparative iTRAQ-based quantitative proteomic analysis was carried out to obtain systematic information of YPM against *B. bassiana* infection.

On the basis of proteomic approaches, immune-related proteins can be detected in certain tissues or organs in insects. For example, a total of 81 immune-related proteins were screened in the *Bombyx mori* proteome, including 32 recognition proteins, 28 signaling proteins, and 21 effector proteins [30]. Meanwhile, in the *Plutella xylostella* proteome, only 58 immune-related proteins were identified [31]. In this study, 132 immune-related proteins were identified, including 46 pathogen recognition proteins, 27 extracellular signal modulation proteins, and 59 immune effectors. In comparison, a greater number of the immune-related proteins were identified from the YPM proteome. However, further functional analyses of the immune-related proteins are required in order to reveal the molecular mechanism of immune recognition that underlies the YPM response to *B. bassiana* infection.

Peptidoglycan recognition proteins (PGRPs) are an important class of pattern recognition receptors, and play a critical role in the innate immune response of insects [32,33]. In addition, some PGRPs are able to recognize structural diversity of peptidoglycans (PGNs) and activate various innate immune pathways, including the Toll pathway, the Imd pathway, and the prophenoloxidase (PPO) activation pathway [34,35,36]. They can initiate downstream immune responses through the recognition and binding of pathogen-associated molecular patterns (PAMPs), thereby inducing the expression of genes that encode antimicrobial peptides (AMPs) [34,37]. For example, the PGRP1 of *O. furnacalis* can recognize the invading microbes and the transcript levels of *PGRP1* increased in response to bacterial and fungal challenges [38]. In *Antheraea pernyi*, the interference of *ApPGRP-B* resulted in a significant increase in the AMP genes in the immune response [39]. In this study, the expressions of PGRP-B (ADU33185.1), PGRP (XP_028160373.1), and PGRP-LB (XP_013143081.1) were up-regulated, indicating that the immune recognition of YPM was activated upon *B. bassiana* challenge. Remarkably, PGRP-B, PGRP, and PGRP-LB possess an N-acetylmuramoyl-L-alanine amidase domain according to the IPR enrichment analysis. The catalytic PGRPs are capable of cleaving the amide bond of bacterial PGNs, thereby exerting direct bactericidal activity [39,40]. However, further study is required to determine whether the PGRPs in this study indeed contain catalytic enzyme activity. Serine proteases (SPs) are important proteolytic enzymes and widely found in insects [41]. Extracellular SPs have been demonstrated to form cascades, and the reaction is rapidly stimulated; meanwhile, the signals of pathogen invasion are amplified [42,43]. Some SPs with one or more disulfide-bridged structures are designated as clips, which indicate the presence of clip domains [42,44]. Clip-domain serine proteases (Clip-SPs) constitute the main members of the extracellular SP cascade pathway in insects and participate in various physiological processes (such as embryonic development and immune responses) [43,45,46]. Studies have demonstrated that Clip-SPs involving the immune cascade pathways can lead to the activation of PPO and the Toll-ligand Spätzle [42,47]. In the present study, a Clip-SP (XP_013184392.1) with up-regulated expression was identified on the basis of the proteome data. Therefore, we speculated that the Clip-SP of YPM may be involved in the activation of the PPO cascade and the Spätzle–Toll pathway. However, due to the limitations of this study, the function of this Clip-SP needs to be investigated further.

AMPs produced and secreted from the fat body are crucial components of humoral immunity [48]. They form a first line of host defense against a variety of potential invaders, including but not limited to bacteria, fungi, viruses, and parasites [49]. In insects, AMPs are generally classified into four groups on the basis of the secondary structures [48,50]. Among them, attacins are large glycine-rich peptides containing a signal peptide, a pro-peptide domain, an N-terminal domain, a conserved motif, and two glycine-rich domains at the C-terminus [48,51]. Previous studies have demonstrated that attacins were active against bacteria and fungi in lepidopteran insects [52,53]. As an example, in *Drosophila melanogaster*, attacin C was activated against Gram-negative bacteria and played a key role in the antimicrobial defense [54]. Similarly, an attacin from *Hermetia illucens* possessed effective antimicrobial activity against the Gram-negative bacteria [55]. In this study, defense protein 3-like (XP_023937619.1) was identified based on the proteome data, which belonged to an attacin C. Given these findings, we speculated that the up-regulation of this protein in YPM after *B. bassiana* infection may imply its potential role in antifungal immunity.

Through GO categories and KEGG pathway analyses, more DEPs were found to be mainly related to metabolic processes and ribosomes. Although there are clear distinctions between the concepts of immunity and metabolism in biological systems, they are interlinked in animal physiology [56]. In invertebrates, the interactions between the immune system and metabolism are an evolutionarily conserved phenomenon [57]. Initiating the immune system is an energy-consuming process. During a pathogenic infection, the immune response is activated, which is related to a systemic metabolic switch that redirects nutrient flow towards the immune system for the elimination of pathogens [56,58]. Meanwhile, a lot of proteins involved in the ribosome were differentially expressed throughout the *B. bassiana* infection, including ribosomal protein L17, L6, S8, and S5, as well as the 60S ribosomal protein and 40S ribosomal protein. It was reported that certain ribosome proteins were associated with cell structure, protein translation, and protein biosynthesis [59,60]. The present results suggested that changes in DEPs may potentially affect protein translation and protein biosynthesis during the *B. bassiana* infection.

## 5. Conclusions

In summary, the protein changes in YPM larvae in response to *B. bassiana* infection were investigated by a comparative iTRAQ-based quantitative proteomic analysis. The immune-related proteins were identified based on the proteome data. The DEPs were screened and identified and then subjected to functional enrichment analysis. These findings will be conducive to further understanding the immune mechanism of YPM and providing relevant information for insect pest management.

## Figures and Tables

**Table 1:** Statistics of protein identification.

Total Spectra	Matched Spectra	Peptides	Identified Proteins	All Quantifiable Proteins
565,469	62,669	29,155	4197	4195

**Table 2:** Statistics of protein difference analysis from the YPM larvae proteome.

Number of Total Proteins	Regulated Type	FC > 1.2	FC > 1.3	FC > 1.5	FC > 2.0
565,469	up-regulated	126	92	57	11
	down-regulated	72	47	13	0

**Table 3:** Differentially expressed proteins of YPM larvae after treatment with *B. bassiana*.

Protein	Description	Accession No.	FC	*p* Value	Type
Cluster-10258.20366; orf1	arylphorin subunit alpha-like	XP_028169947.1	0.62	0.0028	down
Cluster-10258.18909; orf1	protein henna	XP_026753049.1	1.57	0.0043	up
Cluster-10258.20977; orf1	peptidoglycan recognition protein B	ADU33185.1	1.97	0.0171	up
Cluster-10258.20744; orf1	GTP cyclohydrolase 1 isoform X1	XP_028166842.1	1.63	6.01 × 10^−4^	up
Cluster-10258.5049; orf1	elongation factor 1-alpha	XP_022204799.1	1.85	0.0211	up
Cluster-10258.20686; orf1	pancreatic triacylglycerol lipase-like	XP_023935092.1	1.63	0.0258	up
Cluster-10258.39479; orf1	ribosomal L6 and ribosomal S8 and ribosomal S5 C and ribosomal S5 domain containing protein	CDW61069.1	2.15	0.0234	up
Cluster-10258.20398; orf1	tyrosine 3-monooxygenase isoform X1	XP_028171956.1	1.72	0.0136	up
Cluster-10258.20923; orf1	hypothetical protein evm_001044	RVE54217.1	0.61	6.82 × 10^−4^	down
Cluster-10258.14382; orf1	chemosensory protein 2	AHX37219.1	0.63	0.0257	down
Cluster-10258.2265; orf1	heat shock protein 70-4	AQP31364.1	1.56	0.0203	up
Cluster-10258.5046; orf1	hypothetical protein B5V51_858	PCG72390.1	0.65	0.0305	down
Cluster-10258.19866; orf1	phosphoenolpyruvate carboxykinase [GTP]-like	XP_028168927.1	0.65	0.0316	down
Cluster-10258.5607; orf1	serine protease easter-like isoform X2	XP_013184392.1	1.53	0.0136	up
Cluster-10258.17235; orf1	tryptase-like	XP_013193793.1	1.85	0.0448	up
Cluster-10258.20506; orf1	peptidoglycan recognition protein-like	XP_028160373.1	1.65	0.0114	up
Cluster-10258.37244; orf1	60S ribosomal protein L13	XP_008552884.1	2.02	5.25 × 10^−4^	up
Cluster-10258.20313; orf1	uncharacterized protein LOC114360519	XP_028171047.1	1.57	0.0475	up
Cluster-10258.36590; orf1	40S ribosomal protein S4-like, partial	XP_021339550.1	1.62	2.55 × 10^−4^	up
Cluster-10258.36281; orf1	hypothetical protein DDB_G0285741	XP_638067.1	1.64	0.0211	up
Cluster-10258.19803; orf1	chemosensory protein	APG32552.1	0.57	0.0455	down
Cluster-10258.36541; orf1	S10	AAX48886.1	1.86	0.0158	up
Cluster-10258.18280; orf1	uncharacterized protein LOC114364556	XP_028176547.1	1.74	7.80 × 10^−5^	up
Cluster-9364.0; orf1	endocuticle structural glycoprotein ABD-4-like	XP_028173253.1	0.58	0.0124	down
Cluster-10258.27935; orf1	peptidoglycan-recognition protein LB-like	XP_013143081.1	1.94	0.0049	up
Cluster-10258.36298; orf1	uncharacterized protein LOC111678542	XP_023295698.1	1.58	0.0112	up
Cluster-10258.2249; orf1	60S ribosomal protein L2-A-like	XP_021339551.1	1.80	0.0019	up
Cluster-10258.30985; orf1	venom serine carboxypeptidase-like	XP_028155892.1	0.60	6.37 × 10^−4^	down
Cluster-10258.23395; orf1	defense protein 3-like	XP_023937619.1	2.45	0.0072	up
Cluster-10258.2238; orf1	elongation factor, putative	XP_002783366.1	1.69	0.0073	up
Cluster-10258.1655; orf1	ribonucleoprotein, putative	ELP90168.1	2.29	9.19 × 10^−4^	up
Cluster-2690.2; orf2	uncharacterized protein LOC111689114	XP_023307388.1	1.65	0.0198	up
Cluster-10258.36368; orf1	60S ribosomal protein L7-like	XP_028405051.1	1.52	0.0338	up
Cluster-10258.2136; orf1	elongation factor 1-beta-like	XP_022205053.1	1.67	0.0090	up
Cluster-10258.36383; orf1	predicted protein	XP_001625520.1	1.52	0.0025	up
Cluster-10258.37625; orf1	pyruvate dehydrogenase complex dihydrolipoamide acetyltransferase	XP_013761136.1	2.84	0.0303	up
Cluster-10258.2339; orf1	hypothetical protein	AIU94794.1	1.97	0.0024	up
Cluster-10258.36446; orf1	60S ribosomal protein L10-like	XP_015760823.1	2.30	0.0114	up
Cluster-2690.2; orf1	uncharacterized protein LOC111689114	XP_023307388.1	3.72	0.0123	up
Cluster-10258.36809; orf1	40S ribosomal protein S14	KXJ11429.1	2.14	0.0036	up
Cluster-10258.36363; orf1	60S ribosomal protein L5-like, partial	XP_021339565.1	1.87	0.0078	up
Cluster-10258.2314; orf1	stress-70 protein, mitochondrial	RDD38839.1	1.74	0.0143	up
Cluster-10258.27756; orf1	uncharacterized protein LOC114364166	XP_028175991.1	0.60	0.0126	down
Cluster-10258.2302; orf1	guanine nucleotide-binding protein	XP_004343796.1	1.98	3.34 × 10^−5^	up
Cluster-10258.36260; orf1	fatty acid-binding protein-like	XP_022204143.1	1.54	0.0127	up
Cluster-10258.20425; orf1	uncharacterized protein LOC114366599	XP_028179325.1	0.59	0.0242	down
Cluster-10258.36738; orf1	K^+^ channel protein	KJE97207.1	1.72	0.0054	up
Cluster-10258.36471; orf1	40S ribosomal protein S24	XP_013405352.1	1.53	0.0059	up
Cluster-10258.2113; orf1	60S ribosomal protein L25-B-like	XP_022204254.1	2.46	0.0014	up
Cluster-10258.36660; orf1	hypothetical protein pdam_00013747	RMX60612.1	1.54	0.0269	up
Cluster-10258.27291; orf1	putative ferric-chelate reductase 1 homolog	XP_028167929.1	1.52	0.0127	up
Cluster-10258.36259; orf1	transaldolase	OQV22424.1	1.78	0.0086	up
Cluster-10258.37144; orf1	eukaryotic translation initiation factor 5A-1	XP_015794685.1	1.57	0.0052	up
Cluster-10258.2387; orf1	ribosomal protein L17	ABO26685.1	1.56	0.0107	up
Cluster-10258.37453; orf1	trichothecene biosynthesis protein 14 OS	G0KYA7	1.93	0.0096	up
Cluster-10258.37326; orf1	hydroxymethylglutaryl-CoA synthase 1-like	XP_027216736.1	1.55	0.0400	up
Cluster-10258.29648; orf1	hypothetical protein evm_006310	RVE49064.1	1.59	0.0257	up

## Data Availability

The proteome data are available via ProteomeXchange with the identifier PXD063941 (https://proteomecentral.proteomexchange.org/cgi/GetDataset?ID=PXD063941, accessed on 14 May 2025).

## Supplementary Materials

The following supporting information can be downloaded at: https://www.mdpi.com/article/10.3390/insects16070667/s1, Table S1: Liquid chromatography elution gradient; Table S2: Primers used for qRT-PCR in the present study; Table S3: Immune-related proteins identified in YPM proteome.
